# Horizontal transfer of a retrotransposon between parasitic nematodes and the common shrew

**DOI:** 10.1186/s13100-019-0166-3

**Published:** 2019-05-30

**Authors:** Sonja M. Dunemann, James D. Wasmuth

**Affiliations:** Department of Ecosystem and Public Health, Faculty of Veterinary Medicine, University of Calgary, 3280 Hospital Drive NW, Calgary, T2N 4Z6 Canada

**Keywords:** RTE, RTE1_Sar, Horizontal transposon transfer, Host-parasite interactions

## Abstract

**Background:**

As the genomes of more metazoan species are sequenced, reports of horizontal transposon transfers (HTT) have increased. Our understanding of the mechanisms of such events is at an early stage. The close physical relationship between a parasite and its host could facilitate horizontal transfer. To date, two studies have identified horizontal transfer of RTEs, a class of retrotransposable elements, involving parasites: ticks might act as vector for BovB between ruminants and squamates, and AviRTE was transferred between birds and parasitic nematodes.

**Results:**

We searched for RTEs shared between nematode and mammalian genomes. Given their physical proximity, it was necessary to detect and remove sequence contamination from the genome datasets, which would otherwise distort the signal of horizontal transfer. We developed an approach that is based on reads instead of genomic sequences to reliably detect contamination. From comparison of 43 RTEs across 197 genomes, we identified a single putative case of horizontal transfer: we detected RTE1_Sar from *Sorex araneus*, the common shrew, in parasitic nematodes. From the taxonomic distribution and evolutionary analysis, we show that RTE1_Sar was horizontally transferred.

**Conclusion:**

We identified a new horizontal RTE transfer in host-parasite interactions, which suggests that it is not uncommon. Further, we present and provide the workflow a read-based method to distinguish between contamination and horizontal transfer.

**Electronic supplementary material:**

The online version of this article (10.1186/s13100-019-0166-3) contains supplementary material, which is available to authorized users.

## Background

Transposable elements, or transposons, are DNA elements vertically transmitted in eukaryotes, from parents to offspring and ancestral to descendant species. In some cases however, a transposon might be horizontally transferred between different species, that is, transferred between species by other means than vertical transfer. BovB (Bovine-B), a non-LTR retrotransposable element present in ruminants and squamates, has been extensively studied as the first case of a horizontal transfer of a retrotransposable element between eukaryotes [1–3]. Recently, two other cases of horizontal transfers of non-LTR elements have been reported: AviRTE has been detected in birds and human parasitic filarial nematodes including the eye worm *Loa loa* [4], and LINE-1 has possibly been transferred between marine eukaryotes [3]. It has been hypothesized that transposons survive longer if they escape host silencing mechanisms through horizontal transfer [5]. Although the mechanisms of HTTs are still unclear, they could be facilitated by parasites and pathogens [5–7]. Parasites have long lasting, physical contact to their hosts, which increases the chances of HTT either directly or through a secondary pathogen. There have been numerous reports of HTT between parasites and their hosts. In nematodes, in addition to the previously mentioned AviRTE, the DNA transposon *bandit* has possibly transferred between hookworms and humans [8]. In ectoparasites, ticks are the likely intermediate for the spread of BovB [2], but the bed bug (*Cimex lectularius*) and the leech (*Helobdella robusta*) have also been indicated as potential vectors of BovB [3]. The kissing bug (*Rhodnius prolixus*), blood feeder and vector of Chagas’ disease, harbors multiple horizontally transferred transposons: DNA transposons SPIN and OC1 share over 95% consensus sequence identity between the kissing bug and its hosts, opossums and squirrel monkeys [6].

After being horizontally transferred, transposons can have significant impacts on the recipient organisms. They can proliferate in the host genome and increase the genome size significantly. The genome of the strawberry poison frog (*Oophaga pumilio*) is 6.76 Gb large, 4.76 Gb of which are repetitive elements. These include transposons which continued to proliferate after horizontal transfer from other species, possibly lizard and fish [9]. Transposons and their intra-genomic movement can lead to changes in a species’ genotype and phenotype (reviewed by [10, 11]). Transfers of transposons between species have been shown to support the creation of new genes [12]. In tomatoes, insertion of a retrotransposon called Rider into the PSY1 gene leads to a yellow phenotype of the fruit, while duplication of the SUN gene mediated by Rider leads to the elongated fruit shape of Roma tomatoes [7]. Rider was horizontally transferred between the lineages of *Arabidopsis* and tomato plants.

It is important to distinguish horizontal transfer from contamination of genetic material for example during sample collection. This is not a simple task but necessary if we are to understand if and how frequent horizontal transfers occur, and what the involved species are. Contamination has been previously erroneously identified as horizontal transfer in the literature [13]. The close physical and molecular association between a parasite and its host makes determining horizontal transfer a great challenge. We developed a new approach to distinguish between contamination and horizontal transfer that is similar in concept to existing methods. A common strategy to test for contamination is to examine flanking regions of transposon insertions [3]. If the flanking region does not originate from the organism, the transposon is also considered as contamination. Another strategy is to compare the coverage of sequence reads at the transposon and in flanking regions [14]. To remove the factor of genome assembly issues that arise with repetitive regions, we devised a simple strategy to directly determine contamination at the read level. In contrast to genome assemblies, read pairs and long reads are derived from a contiguous strand of DNA. Our approach takes reads that code for an RTE, and identifies the origin of the non-repetitive part of the read pairs or long reads (see Methods).

Given the previously documented horizontal transfers of BovB and AviRTE, the question arises whether RTEs are prone to horizontal transfer between parasites and hosts. We investigated RTEs in 33 mammalian and 10 nematode - free-living and parasitic - species, to identify potential horizontal transfers. We found RTE1_Sar from *S. araneus* and BovB from ruminants in parasitic nematodes, and with subsequent analysis confirmed RTE1_Sar as the only HTT case. BovB has been analyzed previously in multiple studies, but is has not been reported in nematodes: in fact, Ivancevic and colleagues reported BovB explicitly as absent in 12 nematode species [3]. This raised the question of whether the finding of RTE1_Sar in *S. araneus* and BovB in several nematodes resulted from misassemblies due to contamination from/to endoparasites. We developed a simple approach to test for contamination based on long or paired-end reads, and confidently showed that BovB in *H. contortus* is likely derived from a contamination, and that RTE1_Sar is a true endogenous element in the *S. araneus* genome. We tested for horizontal transfer of RTE1_Sar between *S. araneus* and nematodes by estimating a phylogeny and relative RTE1_Sar copy ages. We found that RTE1_Sar was probably horizontally transferred between an unsampled parasitic nematode and *S. araneus* after the split of the lineages leading to *S. araneus* and the hedgehog (*Erinaceus europaeus*, ca. 60 million years ago (mya) [15]).

## Results

### Horizontal transfer of RTEs between parasitic nematodes and their hosts is not common

To detect potential cases of horizontal transfer of RTEs between parasitic nematodes and their hosts, we performed reciprocal sequence similarity searches. We used BLAST [16] to compare 33 mammalian RTEs (Repbase2014 [17]) to 81 nematode genomes (WormBase ParaSite6 [18]), and in addition 10 nematode RTEs to 98 mammalian genomes (RefSeq [19], List of RTEs and genomes in Additional files 1 and 2). To reduce false positives, at least 10 RTE hits were required per species. We detected RTE1_Sar originally described in the genome of the common shrew (*S. araneus*) in nine nematode species, and BovB from cattle (*Bos taurus*) and sheep (*Ovis aries*) in two nematode species (Additional file 3: Table S1). We subsequently tested the different scenarios of horizontal transfer, contamination, and vertical transfer.

### Contamination in the *S. araneus* genome assembly does not confound RTE1_Sar HTT

Contamination of genome assemblies must be considered when searching for HTT. To determine whether nematode DNA, possibly including RTE1_Sar, may have contaminated the library preparation of the shrew genomic DNA, we assessed the general assembly quality and tested for any contamination from other organisms. If the *S. araneus* genome assembly contains other genes from nematodes with RTE1_Sar, it is more likely that RTE1_Sar is a contaminant of the genome.

We used BUSCO [20] to determine the assembly quality of *S. araneus* by testing for the presence of conserved genes. The assembly had a completeness score of 88% (Additional file 3: Table S2), above the average of vertebrates in Ensembl (Additional file 3: Figure S1).

**Fig. 1:**
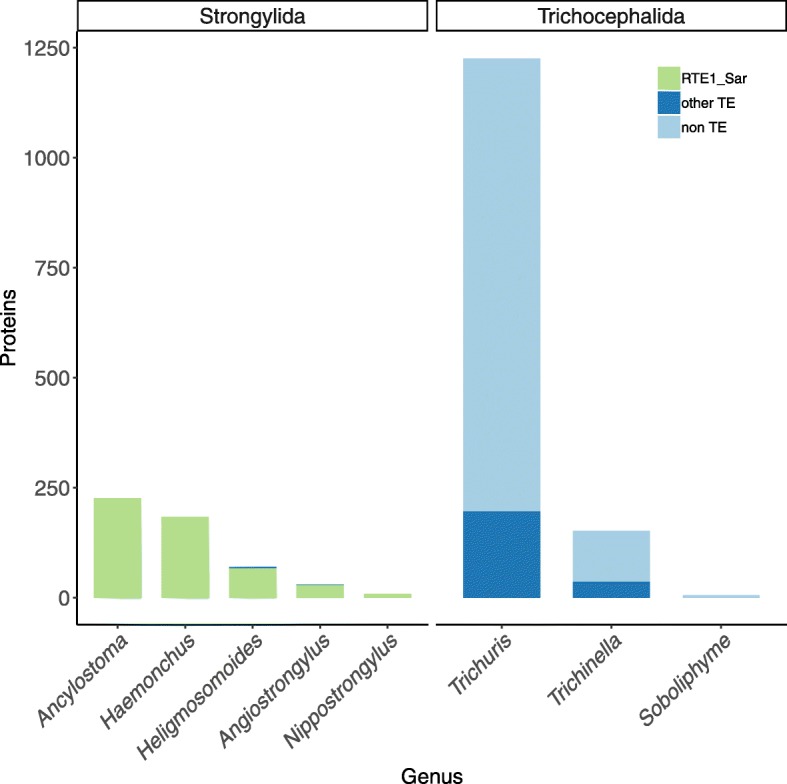
Contamination of the *S. araneus* genome. Reads of the genome were compared with DIAMOND against a UniProt reference database. Hits against nematode proteins were closer examined by annotating the proteins as RTE1_Sar, other TEs and non-TEs by comparing them against Repbase ORFs. Proteins from Trichocephalida are mostly non-TEs from *Trichuris* and *Trichinella*. Four non-RTE1_Sar TEs in Strongylids (dark blue) were annotated as RTE1_Sar when submitted to CENSOR. Graph only shows genera with more than one protein

To identify potential contamination of the *S. araneus* assembly, we assigned taxa of origin to the sequence reads from *S. araneus* with BlobTools [21]. We aligned the sequence reads to the reference assembly with Bowtie2 [22] and compared the reads with DIAMOND [23] against a UniProt reference proteomes database [24]. Of the 2.5 billion sequence reads, 74% mapped to the *S. araneus* assembly. BlobTools estimated that 67% of the aligned reads are from Chordata, and that 7% are of nematode origin (Additional file 3: Figure S2). The nematode species are from the distantly related orders of Trichocephalida and Strongylida (Fig. 1). Over 90% of the reads assigned to the Trichocephalida order mapped non-TE sequences, the rest of the reads mapped to TEs other than RTE1_Sar. In contrast to the reads of Trichocephalida origin, all but 0.8% of the Strongylida aligned reads matched the RTE1_Sar sequence. These 0.8% of reads from *Angiostrongylus* and *Heligmosomoides* aligned slightly better to RTE-5_CPB (*Chrysemys picta bellii*, the western painted turtle), but when submitted to the web-based version of the repeat annotation tool CENSOR [25] - using a different program to screen against repetitive elements - they were annotated as RTE1_Sar. Overall, this finding is consistent with contamination from *Trichuris/Trichinella* genomic DNA, but not with contamination from the Strongylida species.

### Test with long reads and read mates reliably identifies contamination

To further investigate if RTE1_Sar in *S. araneus* or the nematode species results from contamination, we developed a targeted approach that can identify contamination without the need of a well assembled reference genome.

**Fig. 2:**
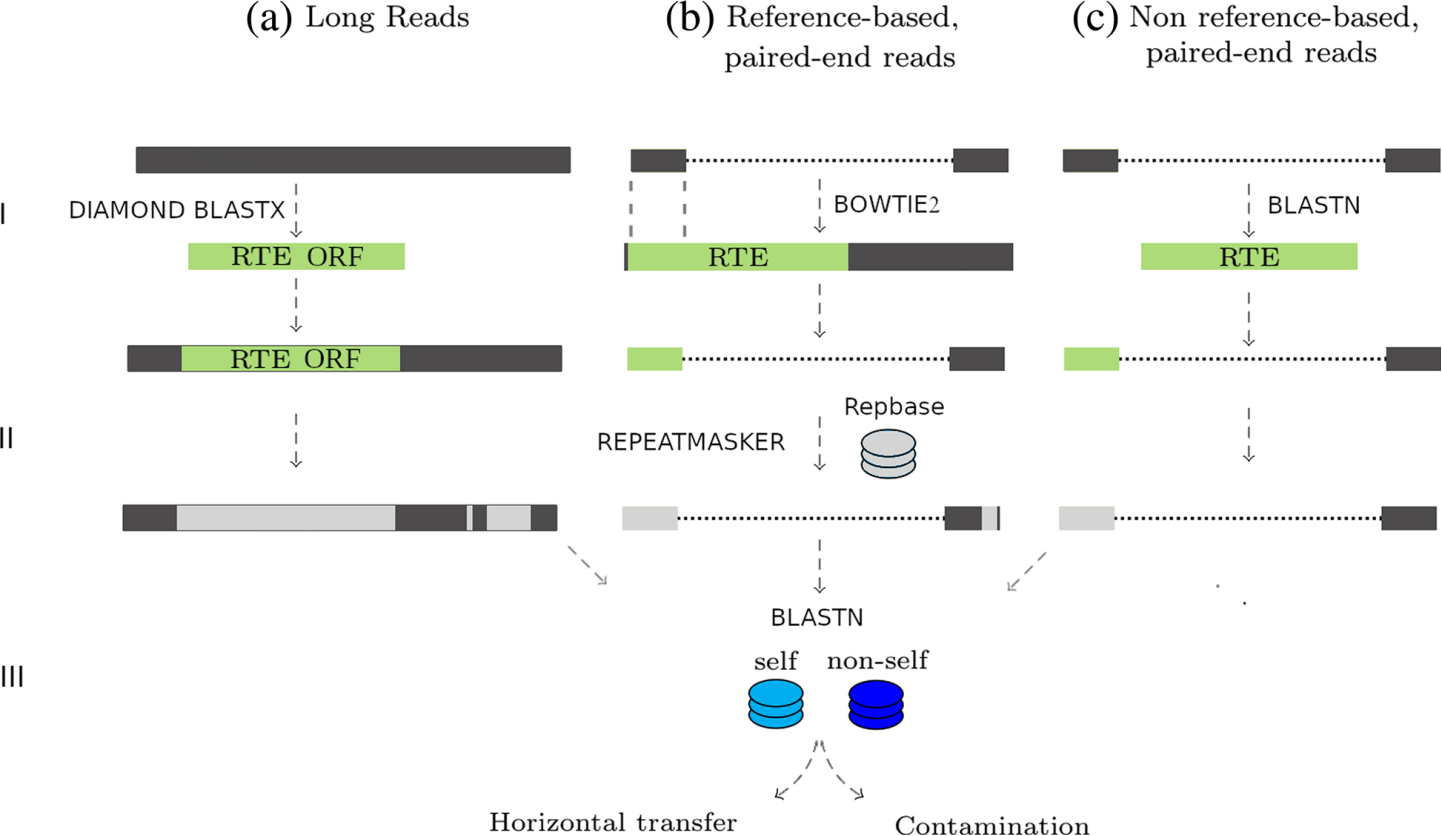
Test for contamination/horizontal transfer with long reads and paired-end reads. 1. Selection of reads that code for a specific RTE. (**a**) Long reads are selected if they code for the RTE, (**b**) paired-end reads are selected if one of the mates maps to a RTE region in the reference assembly, (**c**) paired-end reads are selected if one of the mates codes for the RTE. RTE sequences were previously identified with RepeatMasker in the individual reads and/or the reference assembly. 2. The selected long reads or non RTE-coding read pair mates are compared against two databases. One database (cyan: “self”) contains genomes of the taxon of the tested species, without the genome of the species itself. A second database (blue: “non-self”) contains genomes of the taxon of the species from which the RTE originates. 3. Depending on the comparisons of the reads to the databases, a decision is being made whether the reads containing the RTE belong to the tested species (hit “self” database) and are based on horizontal transfer (cyan), or if the complete long reads/read pairs belong to another taxon (hit “non-self” database) and are based on contamination (blue). Green rectangle: RTE sequence

The idea of our approach is to reliably determine the origin of reads that contain a potential horizontally transferred transposon. This is accomplished by matching the mate of the read, or parts of a long read that do not code for the transposon, to either a donor (“non-self”) or recipient (“self”) database (Fig. 2). If matched to a different taxon, the reads are contaminants. If the reads belong to the tested species/taxa, the transposon has possibly been horizontally transferred.

We developed three different approaches to determine the origin of reads encoding RTEs, depending on whether type of sequence reads and the availability of a reference genome. The three approaches, as shown in Fig. 2, are based on long reads, or short reads with or without a reference assembly. The overall workflow is the same for all three approaches: First step: Identification of RTE coding reads was accomplished by 1) comparison to the RTE sequence directly (long reads and non-reference paired reads), or 2) by alignment to a reference assembly. Second step: Reads coding for RTEs are selected and masked for any repetitive sequences with RepeatMasker. Third step: Comparison of (partially) masked sequences to two databases, “self” and “non-self”. The “self” database includes genomes from the taxon of interest, and the “non-self” database includes genomes from species of potential HTT or contamination.

We used AviRTE as positive control and BovB as negative control after testing it first with the long reads method. PacBio long reads were available for *Haemonchus contortus*, *Loa loa* and Anna’s hummingbird (*Calypte anna*), and Illumina read pairs and reference assemblies were available for *S. araneus*, *H. contortus* and the white-throated tinamou (*Tinamus guttatus*).

We tested if BovB, a transposable element of mammalian origin which we found in *H. contortus* and *O. ochengi*, results from contamination and hence can be used as negative control. We suspected that the observation of BovB in these nematode assemblies was a consequence of contamination with host DNA of sheep (*O. aries*) and cattle (*B. taurus*), respectively. PacBio long reads of *H. contortus* were publicly available. We identified reads with BovB, masked them for repeats with RepeatMasker, and mapped them to the two databases, “non-self” and “self”. Of all BovB-containing long reads, 97% (5309 of 5478) have better hits to mammalian genomes after masking than to nematode genomes. The remaining 3% that mapped against nematode might be the result of contamination of parasite genome assemblies. These results show that BovB transposons in the *H. contortus* assembly are most likely resulting from contamination by the sheep host. The absence of long reads for *O. ochengi* prevented us from carrying out a similar test.

The majority of reads (91.5% reference-based, 85.2% non-reference based) coding for RTE1_Sar in *S. araneus* have better hits to mammalian genomes than to nematode genomes when masked for repetitive elements, indicating that RTE1_Sar is not contamination but native to the *S. araneus* genome. A lower percentage of reads maps to nematodes (8.5% with reference genome, 14.8% without reference). This is most likely based on read mates or parts of the genome that contain very old and fragmented RTE1_Sar sequences, which are not recognized by the pipeline when masking for repeats, and thus in the taxonomy search hit a nematode with similarly fragmented RTE1_Sar sequences.

#### Comparison of methods

**Fig. 3:**
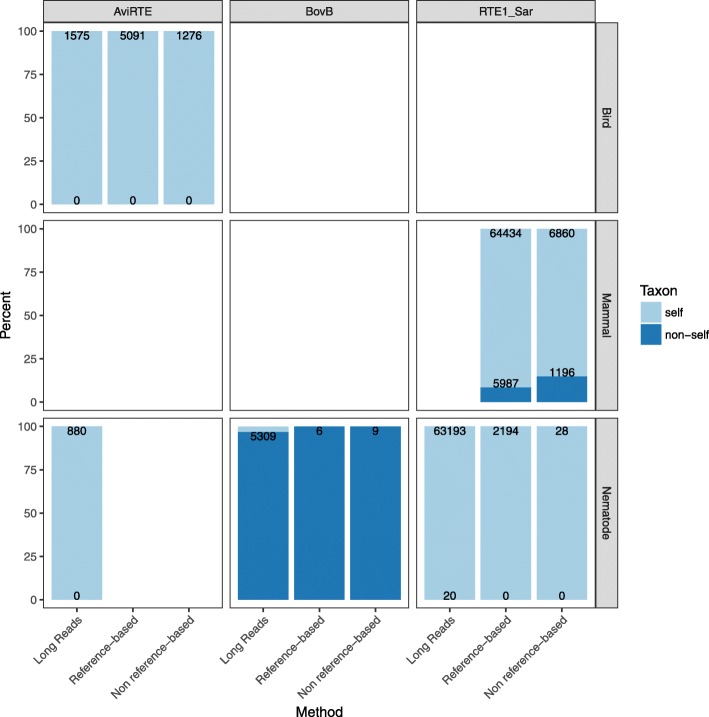
Origin of RTEs in genome assemblies. We used our Snakemake pipeline ‘ConTest’ to test RTE1_Sar is a contaminant in shrews. We used AviRTE and BovB as positive and negative controls. We tested for contamination with AviRTE in birds (short reads of *T. guttatus*, long reads of *C. anna*) and nematodes (long reads of *L. loa*), with BovB in nematodes (short and long reads of *H. contortus*), and with RTE1_Sar in mammals (short reads of shrew) and nematodes (short and long reads of *H. contortus*). Y-axis: percent of reads matching to taxon of origin (“self”) or not (“non-self”), x-axis: method used to identify taxon of read mate. Dark blue suggests contamination, while bright blue does not

All approaches agreed that: 1) RTE1_Sar is not contamination in both *S. araneus* and *H. contortus*, 2) our positive control, AviRTE, is not contamination in either bird or nematode (*T. guttatus/C. anna*; *L. loa*), and 3) our negative control, BovB in *H. contortus*, is contamination (Fig. 3). This shows that these approaches can be used to distinguish between horizontal transfer and contamination. The approaches differ however in the number of informative reads. In all but one case, BovB in *H. contortus*, the reference method reports more reads than the non-reference method. The non reference-based method results in the lowest number of informative reads, which makes it only suited for larger data sets and the reference-based method the preferred approach if long reads are unavailable.

### RTE-1_Sar likely originates from nematodes

We investigated the origin of RTE1_Sar, a transposon originally found in and named after *S. araneus*. We found RTE1_Sar in nine nematode species, all members of the Trichostrongyloidea superfamily. Within mammals, we only found it in *S. araneus*. We have previously shown that misassembly of the genome can be responsible for the perceived absence of a gene [26], which can also be the case for a transposon if only few, old or fragmented copies exist. Therefore, we searched additionally with DIAMOND blastx for the ORF of RTE1_Sar in the short sequence reads of all other species of the order of Eulipotyphla for which genome projects were available: the European hedgehog (*Erinaceus europaeus*), the Hispaniolan solenodon (*Solenodon paradoxus woodi*) and the star-nosed mole (*Condylura cristata*), each representing a different family within the Eulipotyphyla. We found no significant hits (e-value below 1e-10, sequence identity above 75%) in any of these species.

This confirms that the taxonomic distribution of RTE1_Sar spans multiple nematode species with divergent host specificities and only one mammal, suggesting that RTE1_Sar is of nematode origin.

### RTE1_Sar was likely horizontally transferred between parasitic nematodes and *S. araneus*

We compared sequence identities of RTE1_Sar across species, and estimated the phylogeny to test for horizontal transfer. We also tested for neutral evolution of RTE1_Sar copies within genomes to reject a scenario of vertical inheritance, in which the similarity of RTE1_Sar was conserved through purifying selection. Further, we assessed the RTE1_Sar landscapes within genomes to detect potential replication bursts that typically follow horizontal transfer.

**Fig. 4:**
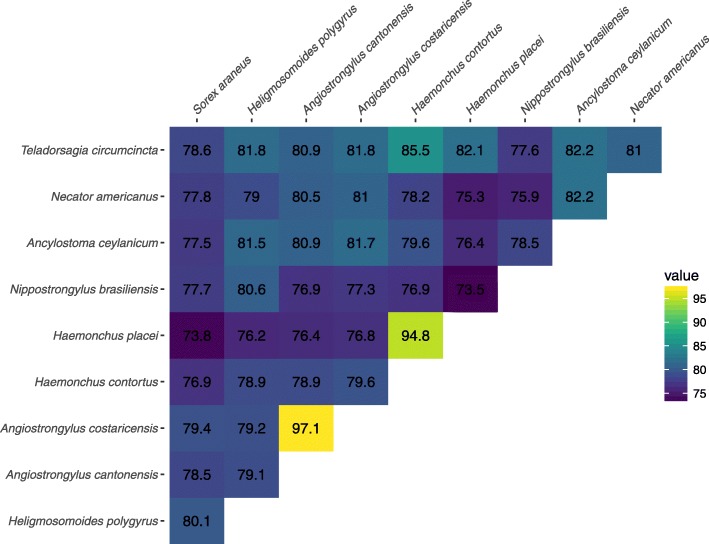
Sequence identities between RTE1_Sar sequences. The sequence identities were measured by pairwise comparisons of full-length RTE1_Sar consensus sequences. RTE1_Sar of *S. araneus* is depicted in the first column, and shares highest sequence identity with *H. polygyrus*

To compare sequence identities between nematode and *S. araneus* RTE1_Sar sequences, we first constructed the species-specific consensus sequences. For this, we aligned RTE1_Sar fragments previously detected with blast for each species with MAFFT, and built the consensus sequences with nhmmer’s [27] hmmbuild and hmmemit. We then aligned the species-specific consensus sequences to each other with MAFFT. Figure 4 shows the sequence identity matrix with pairwise sequence identities. RTE1_Sar from *S. araneus* shares highest sequence identity with *H. polygyrus* (80.1%, Fig. 4). This sequence identity is about the same within nematodes. This suggests that a potential horizontal transfer happened between *S. araneus* and an unsampled parasitic Strongylid. Potential mechanisms are discussed later.

**Fig. 5:**
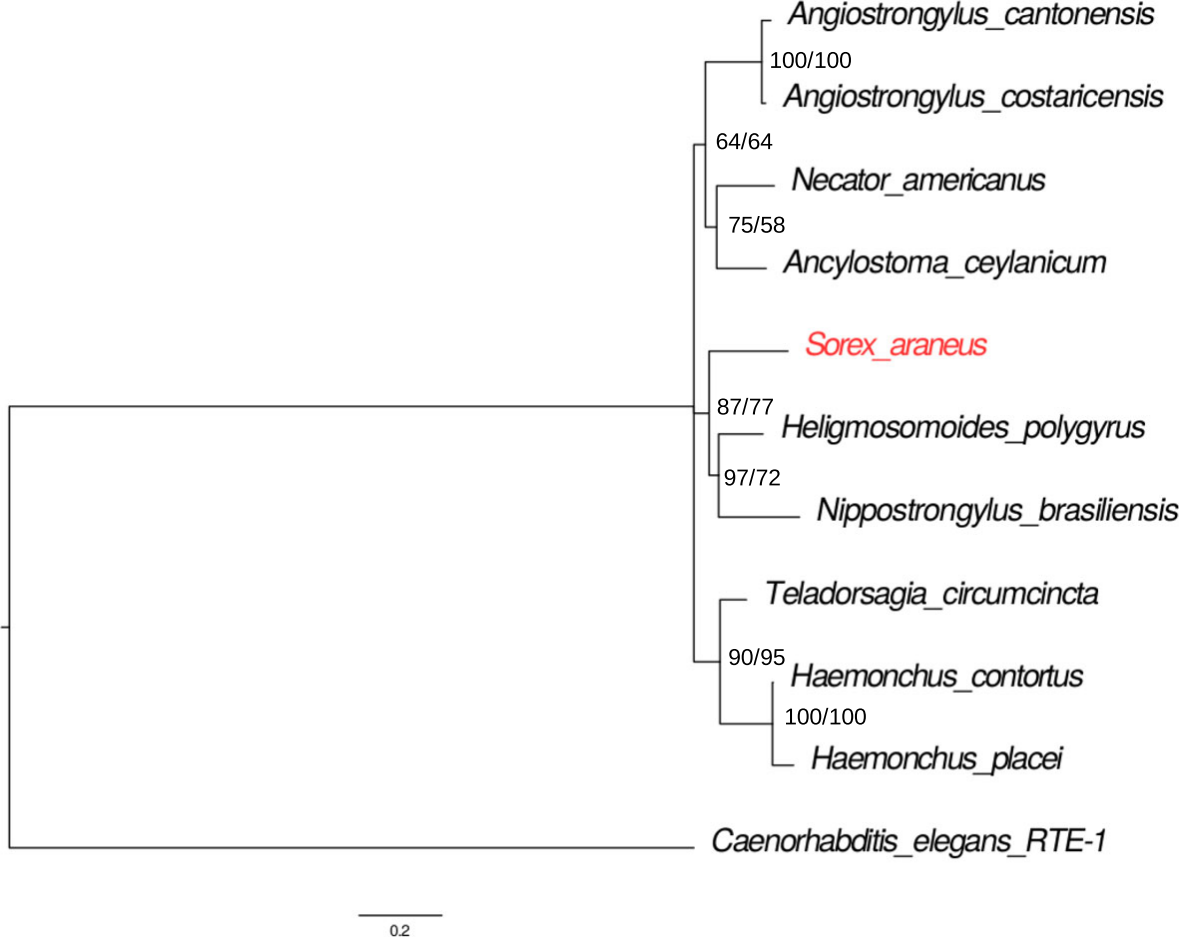
Phylogeny of RTE1_Sar in nematodes and *S. araneus*. Phylogeny was estimated for the ten species that had more than ten copies of RTE1_Sar and suggests a horizontal transfer event. *C. elegans* RTE-1 was used as outgroup. Values at nodes represent support values from MrBayes and RAxML

We used Baysian and maximum likelihood approaches to estimate the RTE1_Sar tree with MrBayes and RAxML, respectively (Fig. 5). We included RTE-1 from *C. elegans* as outgroup and rooted the trees at midpoint. *S. araneus* RTE1_Sar is buried deeply within the nematode RTE1_Sar tree. It is the sister clade to RTE1_Sar in *H. polygyrus* and *N. brasiliensis*. Building the tree based on maximum likelihood resulted in the same grouping of *S. araneus* to *H. polygyrus* and *N. brasiliensis*. The confidence of the location of this clade compared to the other two clades is low. We also estimated maximum likelihood phylogenies based on the amino acid sequences of the open reading frames (ORFs). The ORF of the consensus sequence of *S. araneus* was not full length, so we also took the longest ORF identified from all *S. araneus* copies, and the RTE1_Sar ORF from Repbase. The phylogenies with the fragmented *S. araneus* ORF place *S. araneus* closer to *N. brasiliensis* then to *H. polygyrus*, but the tree based on the Repbase ORF agrees with the nucleotide trees on the position of *S. araneus* (Additional file 3: Figure S3). To not only rely on the consensus sequences, we also used individual copies of RTE1_Sar. We identified individual copies with the respective consensus sequence and RepeatMasker. We used a strict OneCodeToFindThemAll [28] search to concatenate fragments into copies (for copy numbers see Additional file 3: Table S1). The 100 longest and least divergent copies were selected for multiple sequence alignment and tree building with RAxML (GTRCAT model, 1000 bootstraps). The resulting tree places *S. araneus* again close to *H. polygyrus* and *N. brasiliensis* (Additional files 3 and 5: Figure S4).

To evaluate the evolutionary pattern of RTE1_Sar copies within genomes, we aligned the top 100 RTE1_Sar copies for each species separately. These resulted in star-like phylogenies that follow a pattern of neutral evolution (Additional file 3: Figure S5, [12]), suggesting that copies were inserted into the genome and then evolved neutrally, accumulating mutations over time.

**Fig. 6:**
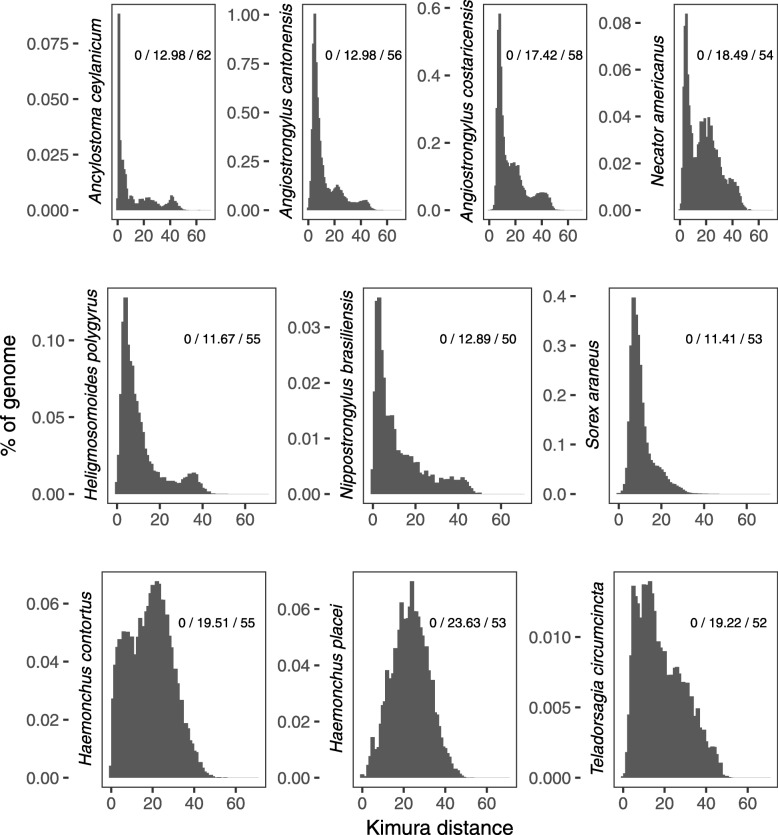
Relative ages of RTE1_Sar copies in their host genomes. The landscape plots represent the divergence of single copies to the consensus sequence (x-axis) and abundance (y-axis). Numbers in plots represent minimum Kimura distance/weighted average Kimura distance/maximum Kimura distance

To assess the RTE1_Sar repeat landscape, which shows the amount and relative age of RTE1_Sar copies, we calculated the Kimura distance of the individual copies to their consensus sequence with RepeatMasker. The shorter the distance, the younger is the age of the copy. The landscapes are similar within each of the three phylogenetic clades, suggesting similar histories of the copies (Fig. 6). The first clade, including both *Angiostrongylus* species, *Ancylostoma ceylanicum* and *Necator americanus*, shows multiple small replication bursts over time, with increasingly young copies. The second clade, including *S. araneus*, *N. brasiliensis* and *H. polygyrus*, have a more gradual increase of copies over time. Interestingly, there is no ancient replication burst in *S. araneus*, but similar to the first clade, *H. polygyrus* has a small ancient replication burst, which is younger in *H. polygyrus* than in the first clade. The third clade has a higher amount of older copies. The average weighted Kimura distance is smallest in *S. araneus*, while the species with the smallest maximum age is *N. brasiliensis*. However, Kimura distances are not directly comparable due to differences in mutation rate and generation time, and due to the lack of clear ancient replication bursts and/or copy age differences this analysis did not confirm the direction of the transfer.

The taxonomic distribution, intra-species copy phylogenies and the consensus tree suggest that RTE1_Sar was horizontally transferred between *S. araneus* and a strongylid nematode, most likely a parasite of *S. araneus* or its ancestor.

## Discussion

HTTs occur frequently in metazoa, with 2772 reported cases on HTT-DB [29]. Some species are more prone for HTT than others, such as parasites that might facilitate HTTs [5, 7]. Two cases of horizontal retrotransposon transfer include parasites either directly (AviRTE) or as a vector (BovB). Here, our aim was to identify potential horizontal RTE transfer between parasitic nematodes and their hosts in order to better understand the frequency of HTT between hosts and parasites. Out of 43 RTEs compared across 197 genomes, we identified a third RTE, RTE1_Sar, that has been horizontally transferred between parasites and their hosts. When testing *Drosophila* species and their wasp parasites, Ortiz et al. did not find strong evidence of HTT between parasites and their hosts [30]. Instead, they found horizontal transfer between different *Drosophila* species. In our study, only a low amount of nematode RTEs were available, which were classified as RTEs from *Caenorhabditis* and *Pristionchus*, which are free-living nematodes. In contrast to the study of Ortiz et al., we found one horizontal transfer between host and parasite, and no cases of HTT between free-living species.

Distinguishing between horizontal transfer and contamination is not easy. Analyses in the tardigrade [13] over-turned earlier suggestions of horizontal gene transfer. We identified RTE1_Sar in parasitic nematodes, and have shown with subsequent analysis that it is not a contaminant in either nematodes or shrew. We designed a protocol to differentiate between contamination and horizontal transfer with long and short reads. The guaranteed continuity of long reads is more reliable that a region assembled from short reads. In the absence of long reads for *S. araneus*, we used paired-end short reads and validated them with PacBio long read libraries from *H. contortus*, *L. loa* and *C. anna*, including positive and negative controls. Our paired-end reads approach provides the same answers as the long read approach, which demonstrates it is a reliable method to test for contamination when no long reads are available.

We exclude a scenario of vertical inheritance and purifying selection coupled with loss of the TE in all other species than the ones included in this study. RTE1_Sar shows an extremely patchy taxonomic distribution, being only present in one mammal and nine parasitic nematodes. In a scenario of vertical inheritance, we would expect to see the transposon in more mammalian species, especially since it is highly abundant in the shrew. However, neither did we detect RTE1_Sar in the genomes of any other mammal, nor in raw reads of close relatives of *S. araneus*. In addition, to conserve the sequence similarity, there must have been purifying selection. We have shown that RTE1_Sar is evolving neutrally, and thus rule out the possibility of vertical transfer. *S. araneus* shares the highest sequence identity of RTE1_Sar with *H. polygyrus*: their consensus sequences are 80.1% identical. The sequence identity between *H. polygyrus* and its closest related species included in the analysis, *N. brasiliensis*, is 80.6%. This suggests, together with the phylogenetic analysis, that RTE1_Sar was not transferred between *S. araneus* and one of the included nematodes, but that an unsampled nematode species was involved. This unsampled species could be an extant species related to *H. polygyrus* and *N. brasiliensis*, or a common ancestor. While all of the nematode species with RTE1_Sar are parasites of mammals, *N. brasiliensis* and *A. cantonensis* have been found in the asian house shrew *Suncus murinus* [31], which, with *Sorex*, is a member of the family Soricidae. Rats are the common host of *A. cantonensis*, *A. costaricensis*, *N. brasiliensis* and *H. polygyrus*, which does not exclude parasitism of shrew, as seen in the case of *N. brasiliensis*. The other species included in this study are parasites of hamsters (*A. ceylanicum*), humans (*A. ceylanicum*, *N. americanus*) and ruminants (*H. contortus*, *H. placei*). Snails and slugs are the intermediate host of *A. cantonensis* and *A. costaricensis*. Although both *Angiostrongylus* species have high RTE1_Sar copy number and sequence similarity to *S. araneus* RTE1_Sar, phylogenetic analysis on both nucleotide and amino acid level suggests that RTE1_Sar in *S. araneus* is closer related to the *Nippostrongyloides*/*Heligmosomoides* lineage. Three species of the *Longistriata* genus, which is closely related to *Heligmosomoides*, have been recorded as parasites of *Sorex spp.* [32]. Another parasite of *S. araneus* is *Soboliphyme soricis* [33], which is closely related to *Trichinella* and *Trichuris* and could explain the contamination we have observed in Fig. 1.

The most parsimonious solution of the horizontal transfer of RTE1_Sar is a direct transfer between *S. araneus* and an unsampled nematode. In this case, the sequence identity between those species would be much higher than the observed sequence identities between the sampled species. In a different scenario, the transfer could have happened indirectly, facilitated by a vector such as a virus or a bacterium. Gilbert et al. demonstrated that transposons can transfer from insects into virus genomes [34]. Other suggested vectors are endosymbiontic bacteria such as Wolbachia [4, 5], which infect a number of filarial nematodes and insects, but none of the species described here.

The taxonomic distribution of RTE1_Sar, supported by the phylogeny would suggest the direction of the transfer from nematodes to an ancestor of *S. araneus*. We were unable to detect the element in the most closely related sequenced species to *S. araneus*: hedgehog, solenodon and star-nosed mole; but it is present in nine nematode species. A previous study showed that RTE1_Sar is more closely related to insect and nematode RTEs than to mammalian RTEs [4]. Copies in the *S. araneus* are highly abundant. Although not proof, this is consistent with horizontal transfer from nematodes and subsequent replication as opposed to vertical inheritance and loss in the other species. As for timing, the transfer must have occurred after the split of the lineages leading to *Sorex* and *Erinaceus*, since we did not detect the element in *E. europaeus*, the closest related species to *S. araneus* with a sequenced genome. The split of the lineages occurred ca. 60 million years ago (mya) [35]. The uncertainty of the direction of the HTT and thus the origin of the element also shows that the transposon annotation in Repbase does not necessarily represent the species or lineage of origin, but the species in which the transposon was first identified. This needs to be considered in future studies, especially those investigating horizontal transfers.

We have analyzed the relative ages of RTE1_Sar copies across all species to describe the RTE1_Sar landscape and to discover potential replication bursts that might follow horizontal transfers. We did not observe sudden replication bursts of older copies in the shrew. Replication bursts indicate a horizontal transfer: genomes newly exposed to a transposon would not have any specific defense mechanisms, which allows for faster replication. The absence of replication bursts could be explained by pre-existing defense mechanisms from similar transposons.

The mechanisms of horizontal transposon transfer are still unclear, but there is a higher chance of HTT between physically or spatially connected species, such as parasites and their hosts. The study finding the highest amount of HTTs so far was conducted in insects, and found that transposons are more likely to be transferred to species sharing closer habitat space [36]. We are still lacking truly comprehensive studies encompassing possible transmission routes between classes or even kingdoms. Venner et al. suggested the use of networks to find transposon transmission routes between interacting species, including parasites and pathogens [37]. Both viruses and endosymbiontic bacteria have been suggested as vectors [4, 5, 30, 38]. With respect to nematodes, horizontal gene transfers from prokaryotes have been identified [39–41]. Beneficial plant cell wall-degrading enzymes were transferred from prokaryotes to plant parasitic nematodes [39]. By knowing the organisms involved in HTT, including parasites and pathogens, we will be able to narrow down possible transfer mechanisms.

## Conclusion

By understanding horizontal transposon transfers we get one step closer to understanding host-parasite interactions and their consequences on genome evolution. Here, we demonstrated that RTE1_Sar has likely transferred from parasitic nematodes to *S. araneus*, and we presented a new method to distinguish contamination from horizontal transfer. We confirm, in addition to studies of BovB and AviRTE, that RTEs can jump between species in close associations such as parasites and their hosts. More studies are needed to estimate the frequency of horizontal transfers, and to investigate potential effects on the new host.

## Methods

### RTE detection in genome assemblies of nematodes and mammals

We screened for nematode RTEs in mammals and for mammal RTEs in nematodes with reciprocal similarity searches to identify potential cases of horizontal RTE transfer between these two taxa. We downloaded 10 nematode and 33 mammal RTEs from Repbase Update (version 21.04.14) [17]. We obtained all representative mammalian genomes from RefSeq [19] (98 total), and nematode genomes from WormBase ParaSite6 [18] (81 total). We compared the RTE sequences to the genomes with BLASTn [16] (v2.4.0+, blastn -evalue 1e-10), and filtered the results using a length cutoff of 100bp. We extracted the genomic locations of each hit from the genome using Bedtools v2.24.0 [42] while filtering duplicates, and used BLAST to extract the nucleotide sequences. We performed a best hit reciprocal similarity search to compare the extracted sequences back to the Repbase database.

### *S. araneus* genome quality and contamination

To test for genome completeness, we quantified the number of conserved genes present in the assembly of *S. araneus* (SorAra2.0, GCA_000181275.2) using BUSCO v3.0.2 [20]. We used BlobTools v1.0 [23] to detect contamination in the *S. araneus* genome. We used Bowtie2 v2.3.3.1 [22] to align sequence reads to the genome (default settings). The sequence reads were downloaded from the NCBI SRA (Bioproject PRJNA13689). To determine the contaminating taxons, we compared the genome assembly against a UniProt reference proteomes database from November 2017 using DIAMOND v0.9.10 [23] with parameters as described in the BlobTools manual (diamond blastx –max-target-seqs 1 –evalue 1e-25 –sensitive).

### Snakemake workflows to determine the taxon of origin of RTE encoding sequence reads

We developed Snakemake [43] workflows to determine whether sequence reads with RTEs originated from the sequenced organism or from contamination. The workflows identify reads coding for a given transposon, and compare the non-repetitive parts or read mates to two respective databases. We publish three different protocols, one for long reads and two for paired-end reads, which are available on github (https://github.com/sdune/contest). Below, we describe the workflows as we used them for this study. For an overview of the workflows, see Fig. 2.

**Long reads.** We downloaded PacBio long reads for *H. contortus*, *C. anna* and *L. loa* (Bioprojects PRJEB2252, PRJNA289277, and PRJNA246086). We downloaded the ORF sequences of RTE1_Sar from *S. araneus*, AviRTE_CAn from *C. anna*, and BovB from *B. taurus*) from Repbase. We then used DIAMOND to find the reads that have high similarity to RTE ORFs (diamond blastx –more-sensitive). The fasta sequences of reads with hits with e-values below 1e-10 were extracted from the fastq files with seqtk v1.2-r94 [44] and masked for transposons with RepeatMasker against all Repbase entries to avoid spurious hits downstream due to repetitive elements. The reads were then blasted (blastn -evalue 1e-10) against two databases to find the origin of the read pair. The databases consisted of the genomes from nematodes and mammals (as described above) or birds (RefSeq, *n*=97) respectively, and the blast parameter for database size was adjusted to the largest genome of the two compared databases. The hits were ranked by bitscore. Since genomes were assembled from reads and thus contain the reads of their own species, hits of reads against the species’ own genome were discarded. The best hit for each read was used to specify the origin of the read as either “self” or as “non-self”, depending on if the best hit was against a species of the species’ taxon or against a species of a different taxon. For example, if the read of a nematode hits its own genome best, this hit is disregarded. If the next best hit is against a different nematode species, the read is considered as “self” or endogenous and hence a sign of HTT. If the next best hit is against a mammal, it is considered “non-self” or contamination.

**Reference-based, paired-end reads aligned to reference assembly.** We downloaded Illumina paired-end sequencing reads from NCBI for *H. contortus*, *T. guttatus* and *S. araneus* (Bioprojects PRJEB4207, PRJNA212876 and PRJNA13689). We annotated the reference assembly for locations of the respective RTE with RepeatMasker, and produced a bedfile by filtering for alignments with sequence divergence under 20% and longer than 200bp. We aligned the paired-end reads to the genome with Bowtie2 and filtered for reads overlapping the annotated RTE regions with Bedtools intersect. We used Samtools [45] to discard read pairs if both mates overlapped RTE regions, and kept the singletons. The singletons and their mates were screened for transposons in Repbase: singletons reciprocally mapping to the RTE were kept, and their mates masked for all repetitive elements. All mates were then mapped against two databases as described above.

**Non reference-based, paired-end reads without reference assembly.** We downloaded Illumina paired-end sequencing reads from NCBI for *H. contortus*, *T. guttatus* and *S. araneus* (Bioprojects PRJEB4207, PRJNA212876 and PRJNA13689) and filtered the sequences for quality with seqtk (seqtk seq -q20). The filtered reads were scanned with blastn for the respective RTE (RTE1_Sar in *S. araneus*, AviRTE in *T. guttatus*, and 17 BovBs in *H. contortus*. Mates of reads with reciprocal hits that do not contain the respective RTE were searched (blastn -evalue 1e-10) against the two databases mentioned above.

### RTE1_Sar detection in sequence reads of Eulipotyphla

We searched for RTE1_Sar in the sequence reads of three additional Eulipotyphla species. We downloaded the sequence reads of *C. cristata*, *E. europaeus*, and *S. paradoxus woodi* from the NCBI sequence read archive [46] (BioProjects PRJNA74585, PRJNA368679 and PRJNA72447), and the amino acid sequence of the open reading frame (ORF) of RTE1_Sar (RTE1_Sar_1p) from Repbase. We used DIAMOND [23] to identify the ORF in the sequence reads (diamond blastx –sensitive). The results were filtered for sequence identity above 75% and e-values below 1e-10 (same e-value as in initial blast search).

### Phylogeny

We estimated the phylogeny of the RTE1_Sar sequences across species to understand the relationship of RTE1_Sar in *S. araneus* and nematodes. This was achieved in four stages: First, we aligned species-specific RTE1_Sar sequences with MAFFT v7.310 [47] using the accuracy-oriented method “E-INS-i”. Second, we created RTE1_Sar consensus sequences for each species from the species-specific alignments of previously identified fragments with hmmbuild and hmmemit (HMMER v3.1b2 [48]). Third, we aligned the species-specific consensus sequences of RTE1_Sar with MAFFT. Last, this alignment was used to estimate the phylogenetic tree of RTE1_Sar with MrBayes v3.2.6 [49]. We used the GTR model with gamma distribution (default: lset nst=6 rates=invgamma). We ran the MCMC for 12 million generations until convergence, with the potential scale reduction factor (PSRF) close to 1 for all parameters. We used RAxML on the same data set under the GTRGAMMA model with 1000 bootstraps. To construct trees based on amino acids, ORFs were identified in each RTE1_Sar consensus sequence using the NCBI ORFfinder [50]. For *S. araneus*, we could not identify a full-length ORF. Hence, we additionally identified the longest individual copy ORF. We extracted the ORF amino acid sequences and estimated their phylogenies using RAxML with the PROTGAMMAGTR model and 1000 bootstraps. Additionally, we constructed a tree with RAxML based on RTE1_Sar copies. Copies were identified using the species specific consensus sequence for searches with RepeatMasker and subsequent de-fragmentation using OneCodeToFindThemAll [28] (–unknown –strict –fasta). The result was sorted by length (descending) and divergence (ascending), and the top 100 copies were extracted from the fasta files for each species. These copies were then aligned with MAFFT. We did not use an outgroup for the phylogeny, and left the tree unrooted.

### Relative age distribution

We calculated the Kimura 2-parameter distance (excluding CpG sites) using the package calcDivergenceFromAlign.pl from RepeatMasker for all RTE1_Sar copies to their species-specific consensus sequence as estimation of the relative age of each copy compared to the species consensus sequence. For raw values, see Additional file 4. We then plotted the age distributions with R [51].

## Additional files

Additional file 1 List of genomes. (XLS 52 kb)

Additional file 2 List of RTEs. (XLS 9 kb)

Additional file 3 Table S1-2 and Figures S1-5. **Table S1** shows stats of RTE1_Sar in genome assemblies. **Table S2** contains the BUSCO scores for the *S. araneus* genome assembly. **Figure S1** shows BUSCO scores for Ensemble vertebrates, **Figure S2** shows the results of the BlobTools analysis for the *S. araneus* genome assembly. **Figure S3** contains phylogenies of RTE1_Sar based on amino acid sequences. **Figure S4** shows a tree of RTE1_Sar based on individual RTE1_Sar copies. **Figure S5** contains star-like phylogenies of individual RTE1_Sar copies within species. (PDF 1686 kb)

Additional file 4 Individual Kimura distances of RTE1_Sar. (CSV 44.7 kb)

Additional file 5 RTE1_Sar phylogeny. (TRE 77.8 kb)

## Abbreviations

BovB: Bovine-B
HTT: Horizontal transposon transfer
RTE: Retrotransposable element
