# Supplementary material for: Horizontal transfer of a retrotransposon between parasitic nematodes and the common shrew
Source: Mob DNA. 2019 May 30;10:24. doi: 10.1186/s13100-019-0166-3 (PMC6542046; doi:10.1186/s13100-019-0166-3)
Supplement: Supplementary file 3 — Table S1-2 and Figures S1-5. Table S1 shows stats of RTE1_Sar in genome assemblies. Table S2 contains the BUSCO scores for the S. araneus genome assembly. Figure S1 shows BUSCO scores for Ensemble vertebrates, Figure S2 shows the results of the BlobTools analysis for the S. araneus genome assembly. Figure S3 contains phylogenies of RTE1_Sar based on amino acid sequences. Figure S4 shows a tree of RTE1_Sar based on individual RTE1_Sar copies. Figure S5 contains star-like phylogenies of individual RTE1_Sar copies within species. (PDF 1686 kb) [file 13100_2019_166_MOESM3_ESM.pdf]

Supplemental Information

Table S1: RTE1_Sar consensus sequence and copy information. The number of hits represent the number of reciprocal blastn hits to RTE1_Sar from Repbase Update. The consensus sequence was built with nhmmer software from a mafft alignment of the top 100 longest hit regions. ORFs were predicted with the ncbi ORF finder. The copy number was assessed with RepeatMasker and OneCodeToFindThemAll.

Species	accession	hits	consensus(bp)	ORF(aa)	copies
Ancylostoma ceylanicum	PRJNA231479	40	2662	893	784
Angiostrongylus cantonensis	PRJEB493	317	2356	793	14246
Angiostrongylus costaricensis	PRJEB494	155	2279	742	7784
Haemonchus contortus	PRJEB506	48	2790	838	3293
Haemonchus placei	PRJEB509	12	2502	267	1621
Heligmosomoides polygyrus	PRJEB1203	68	2520	838	9061
Necator americanus	PRJNA72135	27	1933	491	1989
Nippostrongylus brasiliensis	PRJEB511	56	2834	897	1483
Teladorsagia circumcincta	PRJNA72569	43	2834	927	1915
Sorex araneus	GCF_000181275.1	44098	3350	430	265858

Table S2: Completeness of *Sorex araneus* genome. BUSCO scores: C:88.1% [S:84.5%, D:3.6%], F:5.0%, M:6.9%, n:303. BUSCO scores are based on expected gene content from conserved genes.

Category	n
Complete BUSCOs (C)	267
Complete and single-copy BUSCOs (S)	256
Complete and duplicated BUSCOs (D)	11
Fragmented BUSCOs (F)	15
Missing BUSCOs (M)	21
Total BUSCO groups searched	303

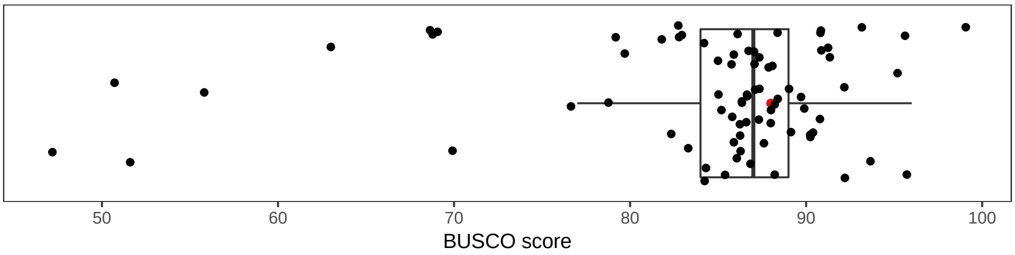

Figure S1: BUSCO scores for vertebrate Ensembl assemblies. Red dot: *S. araneus* assembly.

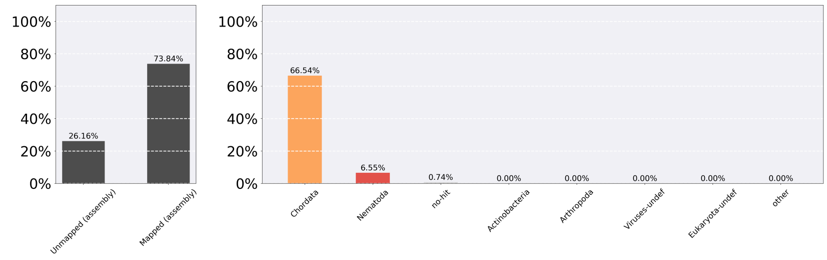

Figure S2: BlobTools analysis for contamination of *S. araneus* genome.

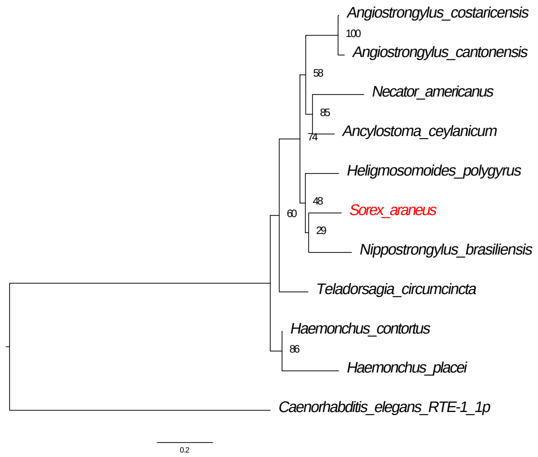

(a) *S. araneus* ORF from consensus sequence.

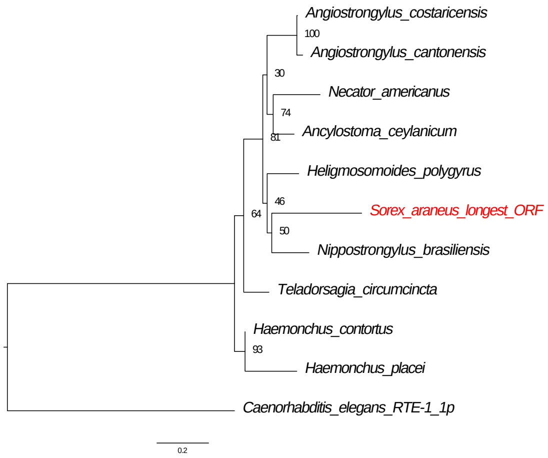

(b) *S. araneus* longest ORF from all individual copies.

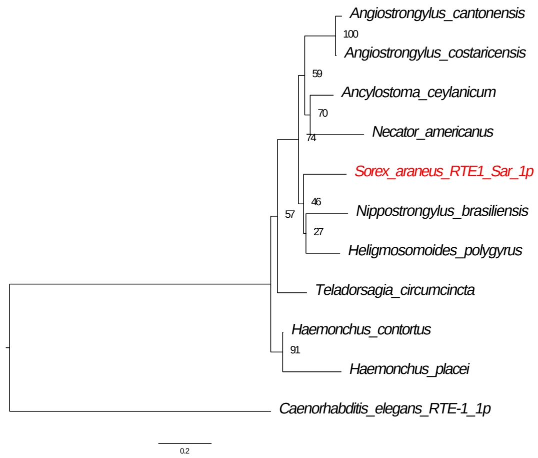

(c) *S. araneus* ORF from Repbase.

Figure S3: RAxML RTE1_Sar trees based on amino acids. Trees are based on ORFs predicted with NCBI's ORFfinder from their consensus sequences, with the ORF of RTE-1 of *C. elegans* from Repbase. We used different ORFs for *S. araneus*: 1) the consensus ORF, 2) the longest ORF from a copy, 3) the ORF from Repbase. The consensus ORF and the longest ORF of *S. araneus* are not full length. Support values are based on 1000 bootstrap replicates.

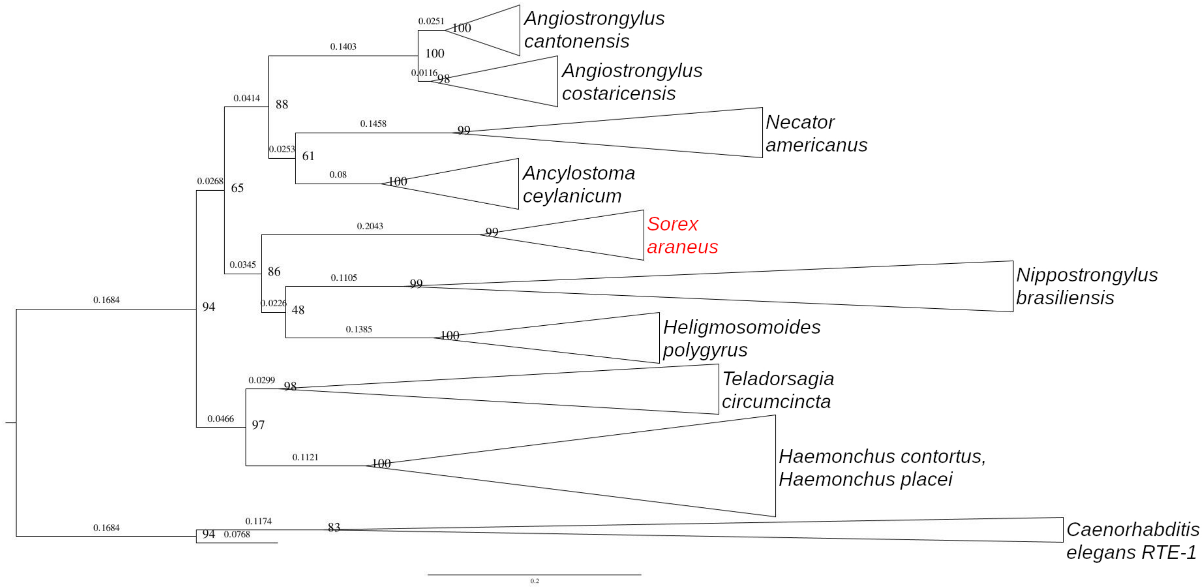

Figure S4: RAxML RTE1_Sar tree based on individual copies. Copies were identified based on the species consensus sequence with RepeatMasker and OneCodeToFindThemAll. The 100 longest and least divergent copies were used for the phylogeny. The tree was rooted with RTE-1 copies of *C. elegans*. Support values are based on 1000 bootstrap replicates.

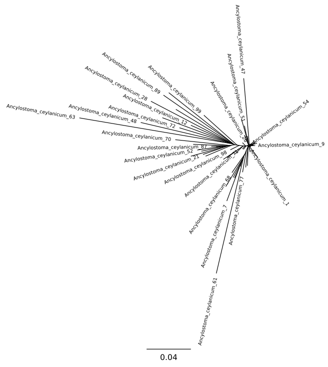

(a) *Ancylostoma ceylanicum*

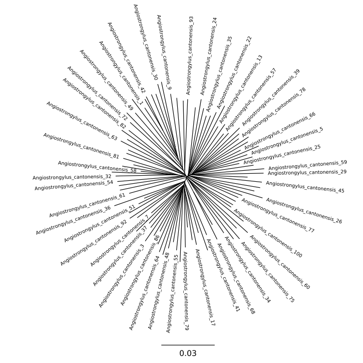

(b) *Angiostrongylus cantonensis*

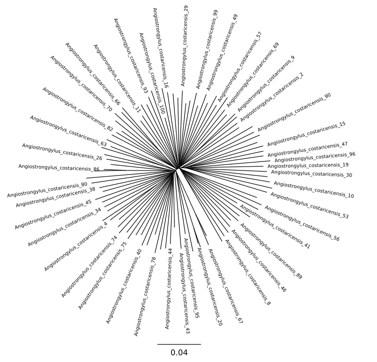

(c) *Angiostrongylus constaricensis*

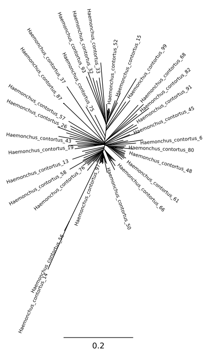

(d) *Haemonchus contortus*

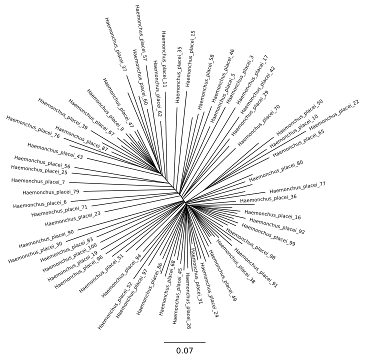

(e) *Haemonchus placei*

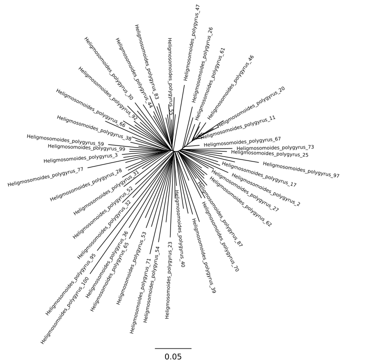

(f) *Heligmosomoides polygyrus*

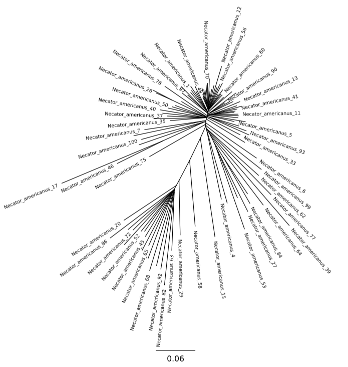

(g) *Necator americanus*

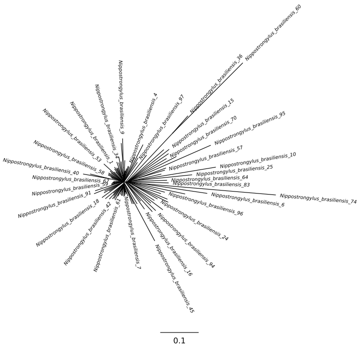

(h) *Nippostrongylus brasiliensis*

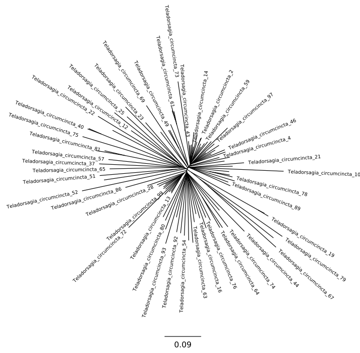

(i) Teladorsagia circumcincta

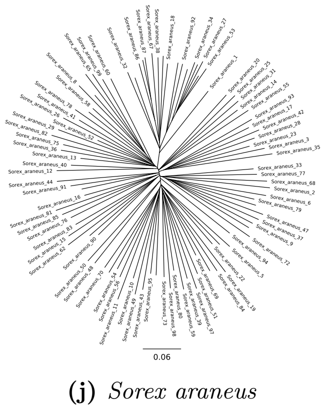

(j) *Sorex araneus*

Figure S5: Phylogenies of species-specific RTE1_Sar copies. Top 100 copies (length, % divergence) were aligned with mafft and phylogenies were built with RAxML GTRCAT. Star-like phylogenies indicate neutral evolution of RTE1_Sar within species.
